# The semi-automatic classification of an open-ended question on panel survey motivation and its application in attrition analysis

**DOI:** 10.3389/fdata.2022.880554

**Published:** 2022-08-11

**Authors:** Anna-Carolina Haensch, Bernd Weiß, Patricia Steins, Priscilla Chyrva, Katja Bitz

**Affiliations:** ^1^Department of Statistics, Ludwig-Maximilians-University Munich, Munich, Germany; ^2^GESIS-Leibniz-Institute for the Social Sciences, Mannheim, Germany; ^3^School of Social Sciences, University of Mannheim, Mannheim, Germany; ^4^Faculty of Economics and Social Sciences, Eberhard Karl University of Tübingen, Tübingen, Germany

**Keywords:** text analysis, support vector machine (SVM), survey methodology, semi-automated analysis, machine learning, survey research

## Abstract

In this study, we demonstrate how supervised learning can extract interpretable survey motivation measurements from a large number of responses to an open-ended question. We manually coded a subsample of 5,000 responses to an open-ended question on survey motivation from the GESIS Panel (25,000 responses in total); we utilized supervised machine learning to classify the remaining responses. We can demonstrate that the responses on survey motivation in the GESIS Panel are particularly well suited for automated classification, since they are mostly one-dimensional. The evaluation of the test set also indicates very good overall performance. We present the pre-processing steps and methods we used for our data, and by discussing other popular options that might be more suitable in other cases, we also generalize beyond our use case. We also discuss various minor problems, such as a necessary spelling correction. Finally, we can showcase the analytic potential of the resulting categorization of panelists' motivation through an event history analysis of panel dropout. The analytical results allow a close look at respondents' motivations: they span a wide range, from the urge to help to interest in questions or the incentive and the wish to influence those in power through their participation. We conclude our paper by discussing the re-usability of the hand-coded responses for other surveys, including similar open questions to the GESIS Panel question.

## 1. Introduction

Open-ended questions in surveys have become more prominent in recent years thanks to the increased use of web surveys. Responses can now be captured digitally, significantly reducing the cost and human effort involved in capturing the responses. However, a primary concern regarding the inclusion of open-ended questions is the increased burden on respondents and researchers. Respondents cannot choose from a pre-defined set of answers but have to access the possible range of answers and choose a suitable answer. From the researcher's perspective, the analysis of responses to open-ended answers requires manual coding, which, when relying solely on human coders, will be costly and impractical, especially if the number of responses is high (Züll and Menold, 2019). Therefore, during the last years, researchers have tried to automate parts of the process with the help of computer-assisted content analysis. This encompasses both dictionary-based as well as supervised machine learning-based procedures (Schonlau, 2015; Schonlau and Couper, 2016; Schonlau et al., 2017; Schierholz and Schonlau, 2021). The later ones are potentially very powerful when mapping respondents' responses to substantially relevant categories, but are yet not widely used in the survey context. *In this article, we will demonstrate how useful supervised learning is for categorizing a large number of responses to open-ended questions, in our case, a question on respondent's motivation to participate in a panel. This article also serves as an illustrative example of how to apply supervised learning in the survey context*. The survey from which we take our data is the GESIS Panel (GESIS, 2021), a German probability-based mixed-mode access panel. In the following, we will describe the pre-processing steps of the text corpus, the semi-automated classification. Since we want to generalize beyond our use case, we will also discuss alternative options regarding the pre-processing and coding steps and look at our semi-automated classification's performance. Finally, we will illustrate the benefit of semi-automated classification by conducting a descriptive evaluation of the respondent's motivation from the GESIS Panel and a more advanced analysis of panel dropout. An evaluation of an open-ended question on panel motivation has not yet been conducted at this granularity; to date, survey motivation has been measured much more coarsely (Porst and Briel, 1995; Brüggen et al., 2011). Therefore, our analysis can provide an unbiased yet clear view of respondents' motivations by combining the openness of the question and the automatic categorization.

## 2. Open-ended questions in survey research

### 2.1. Advantages and disadvantages of open-ended questions

Survey questions that do not provide a set of response options but demand respondents to formulate a response in their own words are known as open-ended questions (Krosnick and Presser, 2010). Open-ended questions are recommended when there is an unknown range of possible answers for the subjects of interest. For instance, we are interested in respondents' motivations to participate in a survey. However, we only have some isolated examples for the possible range of answers, and it is unclear if they are valid for panel participants (in comparison to cross-sectional surveys). Another reason for open-ended questions is an excessively long list of possible answers. An example for such an item with a list of several hundred answer categories would be occupation coding (Schierholz and Schonlau, 2021). Open-ended questions also have the advantage of avoiding being directive in a particular direction through the provided options. Without prompts, respondents have to reflect on the question on a deeper level than choosing a random answer. On the other hand, the need for deeper reflection increases respondents' burden, which can lead to more “don't know” responses or item nonresponse than closed questions do (Krosnick and Presser, 2010). Dillman et al. (2009) recommended using open-ended questions only rarely to not overburden participants. Another reason why open-ended questions are only rarely used is that they also put a burden on the researchers analyzing the data. Analyzing open-ended questions requires the following steps: (1) development of a categorization scheme, (2) coder training, (3) coding, and (4) testing of reliability. Coding can be done either manually or (semi-)automatically. We will move on to these coding options in the next section.

### 2.2. Manual and semi-automated coding

When textual responses to open-ended questions need to be categorized, researchers have two options: manual coding and automatic coding. Manual coding means that a human coder decides which class to assign an answer to, while automatic coding relies upon statistical learning models that assign substantial, a-priory defined categories to textual responses. Manual coding is expensive and time-consuming since it requires human coders; ideally, at least two persons independently code in order to assess inter-coder reliability (Leiva et al., 2006; Schonlau, 2015). Therefore, automatic or semi-automatic coding is an attractive option for large data sets. Completely automatic coding will not be discussed here; the performance quality is, in general, not good enough for the categorization of short texts such as open-ended questions in surveys (Jónsson and Stolee, 2015). However, a subset of the data, called training data, is coded by human coders in semi-automatic coding. A statistical learning model is then trained on this subset. This model is then used to predict the class of uncategorized text responses. Therefore, a disadvantage of semi-automatic coding is the need for expertise to perform the modeling and prediction, but this is offset by lower cost and faster execution (once the modeling is complete). Plus, several applications show that semi-automatic encoding utilizing machine learning algorithms can effectively code and classify different kinds of text data. For instance, Grimmer and Stewart (2013) compared different ways of coding political texts automatically and discussed the advantages and disadvantages of this approach; Gentzkow et al. (2019) did the same for economic data. Open-ended questions in surveys are also just text data, and machine learning algorithms have been used to classify these answers. Kern et al. (2019) give an overview of these applications of statistical learning methods in survey research in general and also discusses open-ended questions (Joachims, 2001; Schonlau and Couper, 2016). Joachims (2001) used a support vector machine (SVM) for the classification of open-ended answers and achieved good performance. Schonlau and Couper (2016) developed a semi-automatic approach where answers to open-ended questions are classified automatically by multinomial gradient boosting when the probability of correct classification is high and manually by a human otherwise. A paper by He and Schonlau (2020) explored using double coded data for classification.

Another practical example of these (semi-)automatic methods for open-ended questions is occupation coding. It refers to the coding of text responses to an open-ended question about the respondent's profession. For example Gweon et al. (2017) proposed three automatic coding algorithms and improved coding accuracy for occupation coding, and Schierholz (2019) compared statistical learning algorithms in occupation coding.

Another example of text data in surveys is responses to exploratory questions, e.g., web probing. Exploratory questions are follow-up questions that ask respondents to provide additional information about a survey item (Beatty and Willis, 2007; Meitinger et al., 2018).

In the following, we introduce our exemplary application area: research on respondents' motivation to participate (or not) in surveys, exemplified using the GESIS Panel. Then, we will explain the central role that survey motivation plays in survey methodological research and give a short overview of previous analyses with data from open-ended questions on survey motivation.

## 3. Collecting and coding of data on survey motivation

### 3.1. Survey motivation

A key concern for panel infrastructures such as the GESIS Panel is maintaining their group of panelists and motivating them to participate in survey waves repeatedly. Even if initial recruitment was successful, throughout multiple panel waves, panel attrition might decrease the number of respondents (Hill and Willis, 2001; Behr et al., 2005; Lynn, 2018), leading to nonresponse bias and variance inflation. Theoretical and empirical research on response behavior has thus been an integral part of survey research for the last decades (Keusch, 2015). Apart from societal level factors such as survey fatigue and attributes of the survey design, respondents' personality traits, topic interest, attitudes toward survey research, and previous participation behavior are examined as a possible influence on response behaviors (Keusch, 2015). Survey motivation is an intermediate step between these external/internal factors and the response behavior. For example, in the frame of the leverage-salience theory (Groves et al., 2000), different survey attributes can have very different effects among possible respondents. The achieved influence of a particular feature is a “function of how important it is to the potential respondent, whether its influence is positive or negative, and how salient it becomes to the sample person during the presentation of the survey request” (Groves et al., 2000, p.301). Although this and other theories (Singer, 2011) of survey participation establish a direct link between survey motivation and survey participation, we know surprisingly little about how people describe their participation motivation when not prompted with pre-defined categories. A short overview of previous research on survey motivation building upon open-ended questions and accompanying classification of survey motivation is given in the next section.

### 3.2. Overview over existing classification schemes

Two studies by Porst and Briel (1995) and Singer (2003) looked at participation motivation in surveys; both use very similar classification schemes to categorize answers to the open-ended question. Singer (2003) included vignettes in a monthly RDD survey and asked respondents how willing they would be to participate in the described survey, and a second open-ended question: “Why would (or Why wouldn't) you be willing to participate in the survey described?.” She then divided the reasons given into three broad categories—altruistic, egoistic, and characteristics of the survey. The author explains that the alleged overlap between survey characteristics and the other two categories is resolved through the respondents' emphasis on themselves and their altruistic motive or the survey characteristics. These categories closely resemble those developed by Porst and Briel (1995) for German panelists. They differentiate three broad categories: altruistic, survey-related, and personal, and develop a classification with finer details ranging from four to seven sub-categories. For example altruistic reasons are divided into the motive “surveys important/meaningful for politics, society, economy, science,” “surveys important/meaningful for ZUMA” (ZUMA was the Centre for Survey Research and Methodology, now part of GESIS), “surveys important/meaningful (without specification),” “social responsibility.” We will employ a similar classification scheme in the analysis of the open-ended question on panel motivation in the GESIS Panel, which we will present next.

## 4. Survey motivation in the GESIS Panel

We use data from the GESIS Panel (Bosnjak et al., 2018), a probability-based mixed-mode panel that has been in operation since 2014. In order to compensate for panelist dropout, there have been two refreshment samples, so that the panel in October 2020 consisted of three cohorts and a total of more than 5,000 respondents (GESIS, 2021). Bi-monthly, panelists are invited to respond to a survey that lasts approximately 20 min. About 75% of respondents answer in web mode and 25% in mail mode. With each survey invitation, they receive a prepaid incentive of five euros.

### 4.1. The question on survey motivation

**Figure 1:**
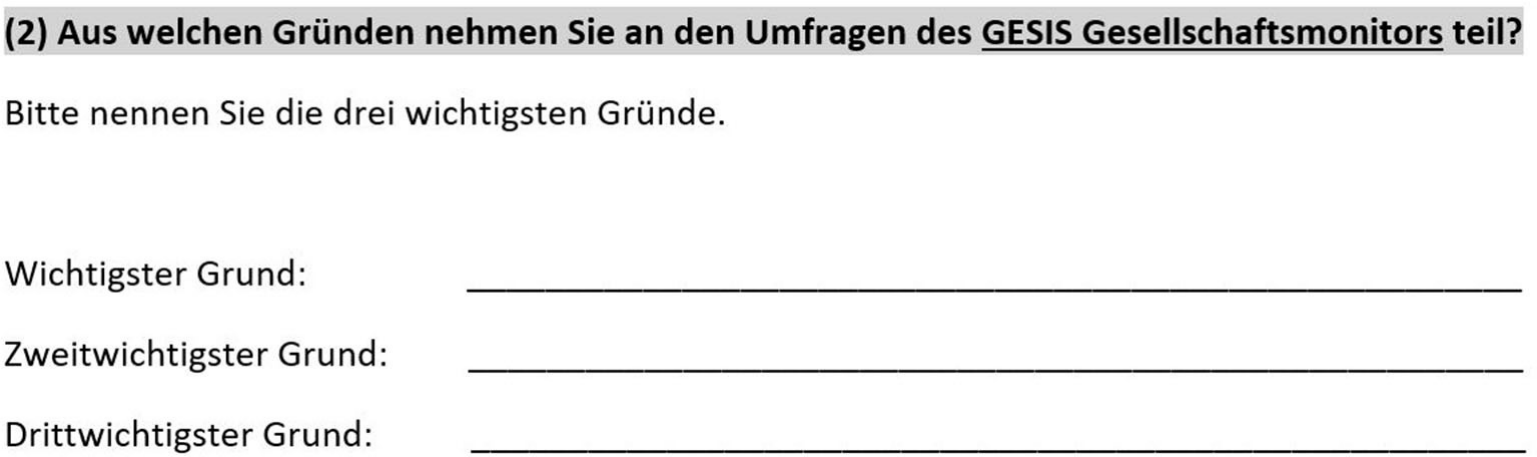
Approximate design of the GESIS Panel questionnaire, mail survey, wave bf, recreated by the authors. Translation reads: “**(2) For what reasons do you participate in the surveys of the** **GESIS GesellschaftsMonitor?** Please name the three most important reasons. Most important reason: …, Second most important reason: …Third most important reason: ….”

Until 2020, the GESIS Panel had six waves per year, and a question on survey motivation was included in every sixth and last wave of a year. Figure 1 shows the design and wording of the survey question. The panelists are asked for what reasons they participate in the GESIS GesellschaftsMonitor surveys. GESIS GesellschaftsMonitor is the name with which the GESIS Panel presents itself to its participants. The panelists are then prompted to give their most important reason, second most important reason, and third most important reason in three separate lines. This questionnaire design has major advantages in coding, since it leads to unidimensional answers with very few exceptions (< 1%).

### 4.2. The semi-automatic categorization of an open-ended question on survey motivation

#### 4.2.1. Manual coding

**Figure 2:**
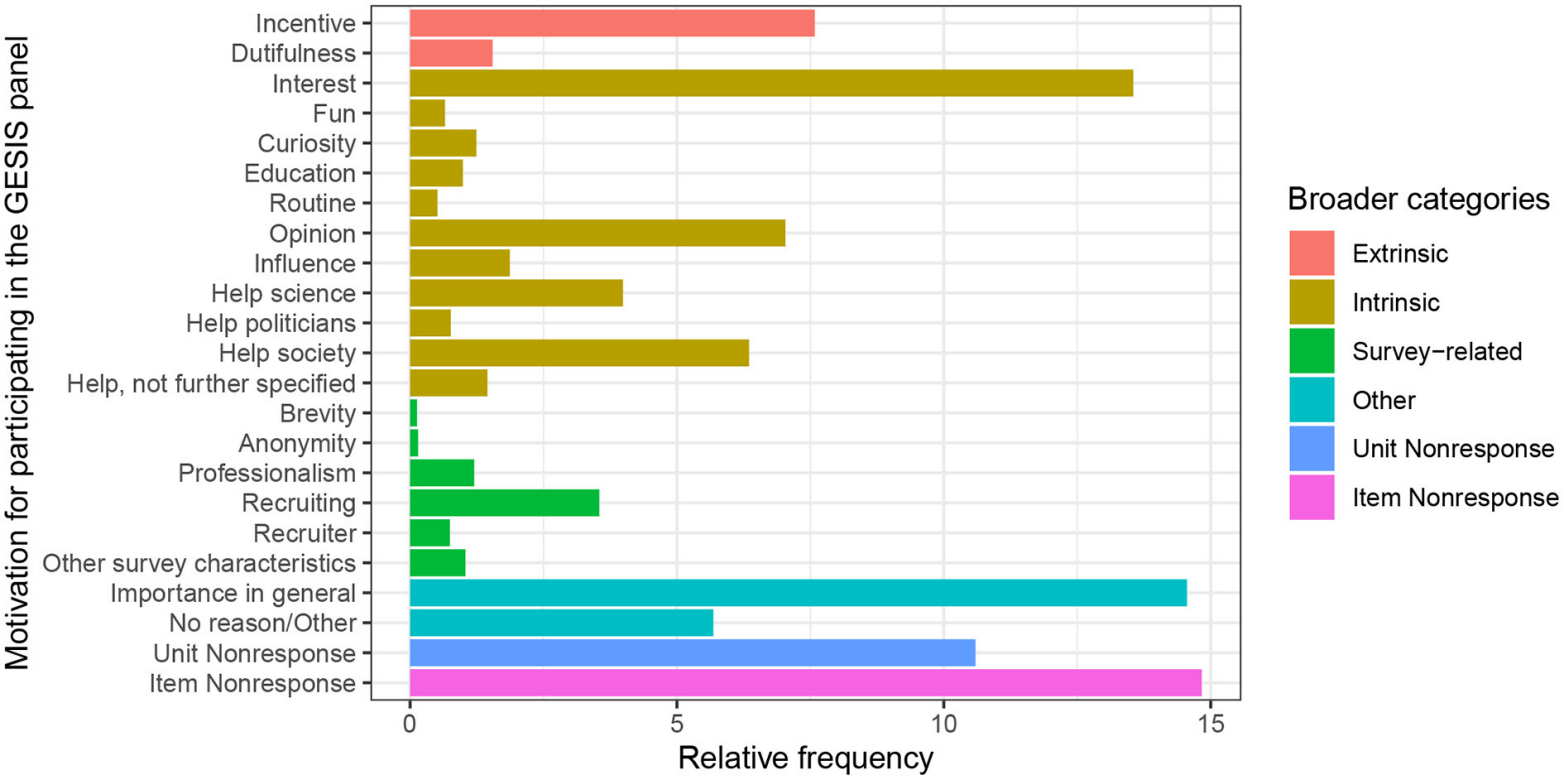
Most important reason given by panelists in wave “bf” (2014) of the GESIS Panel. Semi-automated classification of reasons described in Section 4.2.3.

We first developed our coding scheme before beginning with the manual and semi-automated coding. This was done iteratively by a team of two authors; we started with a coding scheme from Porst and Briel (1995) but adapted it to the GESIS Panel survey, adding categories that were more specific to the GESIS Panel survey and were often mentioned. We aimed for at least 40 observations for each category, collapsing categories that did meet that criterion. We aimed toward categories that were as distinct as possible from other categories. For some of the multivariate analyses, we collapsed the finer categories into broader categories due to sample size. The final list of categories is available in Table 1 and the entire coding scheme (in English and German) in the Appendix. The assignment of finer to broader categories is given in Figure 2. We will give a brief overview of the categories. The first group of possible categories refers to answers that express interest or curiosity in the survey topic. Some respondents indicate that they learn from the survey about current topics or that they learn something about themselves when they reflect and answer the questions (“Learning”). Other respondents participate because they want to share their opinion (“Tell opinion”); some even want to influence policy or research through their participation (“Influence”). Since the GESIS Panel has an incentive of 5 Euro, many people mention it (“Incentive”). Respondents often mention that they enjoy taking the survey (“Fun”) or that they participate because it has become part of their routine (“Routine”). This is, of course, an answer that one would not find in one-time surveys. Some persons feel obliged to participate out of a sense of duty (“Dutifulness”). A lot of people want to help through their participation and they often, but not always, specify the addressees of their assistance: researchers, politicians or even the society in general (“Help science,” “Help politicians” “Help society” and “Help in general”). There were also some survey-related reasons to participate that some respondents mentioned: the brevity, the anonymity, and the professionalism of the GESIS Panel in particular (“Brevity,” “Anonymity,” “Professionalism,” and “Other survey characteristics”). More people than we expected from previous research mentioned their recruitment or even specific traits of their recruiting interviewer for the GESIS Panel. Therefore, we added these categories (“Recruiter” and “Recruitment”). Many respondents simply mentioned that participation in general or the survey are important; this is a comprehensive and common class (“Importance in general”). Some persons cannot think of a reason or give other answers that are very rare, e.g., “I have been pushed to participate by my parents”. These responses are summarized in a residual class (“No reason/Other”). Examples from the survey for each class are available in the Appendix as part of the Coding Scheme.

**Table 1:** List of categories for respondents' motivation to participate in the GESIS Panel.

**Categories**		
1. Interest	10. Help science	19. Other survey characteristics
2. Curiosity	11. Help politicians	20. Importance in general
3. Learning	12. Help society	21. No reason/Other
4. Tell opinion	13. Help	
5. Influence	14. Brevity	
6. Incentive	15. Anonymity	
7. Fun	16. Professionalism	
8. Routine	17. Recruiter	
9. Dutifulness	18. Recruitment	

A more detailed coding scheme with examples can be found in the Appendix.

A random subset of the data (*n* = 5,000), about one-fifth of the data, was manually coded by one of the co-authors and a student assistant independently after two co-authors settled on the final coding scheme. While manually coding 5,000 answers might seem like a very high number in comparison to other studies (Schonlau and Couper, 2016 coded around 500 answers), one should, however, keep in mind that we also used 21 categories. A sufficient number of observations is required for each of these categories. As in many other cases, intercoder disagreement occurred repeatedly (Popping and Roberts, 2009; Schonlau, 2015). We used Cohen's κ-coefficient to calculate the measure of agreement between coders (Fleiss et al., 2003). Cohen's κ-coefficient was high, around 0.91. The remaining disagreements can be resolved in several ways, such as: (1) the two coders discuss the disagreement and reach consensus (D'Orazio et al., 2016), (2) a third person (an expert) with more experience determines the code, or (3) a third coder is used. The third coder can then break the tie between the first two coders. In our case, we resolved disagreements by the second option; the expert was a more senior member of the team of authors.

#### 4.2.2. Pre-processing

Processing and cleaning text data for semi-automated classification can require varying amounts of efforts and techniques, however, a set of typically used techniques has already been established: this set includes spellchecking (Quillo-Espino et al., 2018), lowercasing (Foster et al., 2020), stemming (Jivani, 2011; Bao et al., 2014; Singh and Gupta, 2017), lemmatization (Bao et al., 2014; Banks et al., 2018; Symeonidis et al., 2018; Foster et al., 2020), stopword removal (Foster et al., 2020), and different ways of text enrichment/adding of linguistic features (Foster et al., 2020). We will systematically review these options below and justify our choices (for an overview, see Table 2).

**Table 2:** Steps in semi-automated coding, choice of methods and alternatives.

**Step**	**Options**
**Manual coding of test set**	
Sampling	**Random sampling**, random sampling with min. sample numbers for each class, iterative procedure with coding of observations with low predictive certainty
Number of coders	1, **2**, 3, ... + **additional expert coders to resolve differences in coding**.
Resolving differences in coding	(1) Reaching consensus between original coders (2) **expert decides** (3) majority vote by third coder
**Pre-processing of text data**	
Spellchecking	**Yes**/no
Lowercasing	Yes/**no**
Stemming or	Yes/**no**
lemmatization	Yes/**no**
Stopword removal	Yes/**no**
Tokenization	**Unigrams**, Bigrams, Trigrams ...
Inclusion of word/sentence embeddings	**Yes**/no
Inclusion of non-text data	Yes/**no**
**Semi-automated categorization**	
Statistical learning algorithm	Tree-based methods (e.g., boosting or random forests), **support vector machine (SVM)**, multinomial regression, Naive Bayes classifier
Additional human coding for observations with low predictive probability	Yes/**no**
**Checking/Validation**	
Evaluation parameters	**Accuracy. Precision, Recall, F1, Detection Rate, Detection Prevalence, Balanced Accuracy (either macro or micro). Confusion matrix**.

Our decisions for our example are in bold font.

First, we perform an automated spelling correction through the hunspell R package (Ooms, 2020) using the dictionary “DEde2,” i.e., words that are not part of this German dictionary are replaced with a word that is as similar as possible in spelling to the unknown word. Then, after a first check of the performance of the correction, we expanded the dictionary with words that were incorrectly improved because they were not part of the dictionary but very frequent in our corpus (e.g., “GESIS”). After adding these, two of the authors used a random sample (*n* = 100) to re-check performance and concluded that only about 6% of the improved words were changed so that they no longer corresponded to the meaning that the respondent had intended.

For subsequent pre-processing steps, we used the popular quanteda R package (Benoit et al., 2018), which offers a multitude of text functions like lemmatization and stemming, trimming, upper- and lowercasing. It also allows transforming text snippets into tokens such as uni- and multigrams and also allows functions for computing text statistics, fitting models, and producing visualizations from a text corpus. Text functions such as stemming, lemmatization or even lowercasing reduces the size of the text matrix, making it more dense. Stemming for example is especially desirable for languages where a single stem can generate dozens of words in case of verbs (e.g., French or Turkish). Similarly, lemmatization groups together inflected forms together as a single base form. However, in some cases, the actual word might make a difference, compared to its stem. In our case, differences between, e.g., “I am” and “I was” may carry important information, since referring to the past was often done when referring to the recruiter or the recruiting experience compared to the present used for expressing interest in the questions. We tested both lemmatization and stemming as well as stopword removal, but concluded that not doing either of the steps increased performance. We did remove hyphens, separators and punctuations.

#### 4.2.3. Semi-automated coding: SVM and other options

Semi-automatic text classification is possible through statistical learning. Many statistical learning algorithms are now available in statistical software like R and Python, and it is not possible to give a complete overview here (see e.g., Hao and Ho, 2019, for a Python overview). However, we do want to point to some of the most popular choices that have been applied to classifying answers to open-ended questions: these include tree-based methods like random forests and boosting (Schonlau and Couper, 2016; Kern et al., 2019; Schierholz and Schonlau, 2021), support vector machines (SVM) (Joachims, 2001; Bullington et al., 2007; He and Schonlau, 2020, 2021; Khanday et al., 2021), multinomial regression (Schierholz and Schonlau, 2021) and naïve Bayes classifiers (Severin et al., 2017; Paudel et al., 2018).

From the multitude of possibilities, we explored *via* 5-fold cross-validation with 70% of the hand-classified data the different algorithmic options in the R package LiblineaR (Helleputte, 2021) based on the C/C++ library ‘LIBLINEAR' (i.e., L2-regularized L2-loss SVM, L2-regularized L1-loss SVM, SVM by Crammer and Singer, L1-regularized L2-loss SVM, L1-regularized and L2-regularized logistic regression). Liblinear is able to handle large-scaled data sets, especially for text classification, i.e., data sets where some features are scarce (Fan et al., 2008; Helleputte, 2021). We also explored different cost parameter values (0.1, 1, and 10) combined with these algorithms. We also explored random forests and naïve Bayes classifiers, but they did not yield higher performance rates. After examining the cross-validation (5-fold) results, we chose to use an L1-regularized L2-loss SVM (Liblinear type 5) with cost parameter 10. We retrained the model with this parameter setup and the combined training and test set (70% of the hand-classified data). The accuracy rate for the validation set (30% of the hand-classified data) was 0.93 [0.91, 0.94], and the unweighted median macro F1 measure over all categories was 0.83. We then continued to automatically classify the complete dataset, including the ~20.000 answers not classified by hand. The categories with the weakest performance (F1 measure around 0.6) were Learning, Help in general, and Recruiter. In general, smaller categories or categories that are close to others (e.g., “Help in general” and “Help politicians,” “Help society”) were more difficult to categorize for our trained model. Here, one could also think about collapsing different categories for better performance measures, but also at the cost of being less specific and losing information. Therefore, we decided against this step for our analysis. Larger (and easy to catch) categories such as Interest and Incentive have micro F1 measures of >0.99 in our classification.

### 4.3. Analyses

#### 4.3.1. Univariate analysis of respondents' motivation

We are now moving on to further analysis steps after the semi-automated classification of the not hand-coded observations. First, we are interested in the empirical distribution of reasons given by panelists of the GESIS Panel for their participation. This is important information, especially for panel management and maintenance, as the knowledge of participants' motivation can, for example, be used in further waves for adaptive design measures. In Figure 2, the relative frequencies of the most important reason to participate in the panel, which have been semi-automatically classified, are shown for the panelists of wave “bf” (2014) of the GESIS Panel. As can be seen in Figure 2, the different categories have very unequal relative frequencies. The biggest substantive categories are “Incentive,” “Interest” and “Importance in general.” Many panelists openly state that they are participating because of the 5 Euro incentive given in each wave for participation. Over 10% participate because they are interested in the topics covered in the survey. A substantive part of respondents also state that they participate because they perceive their responses as important, but do not further elaborate on why they think so or for whom they think their participation is important. All in all, around 10% of the panelists state that they want to help, and usually, they also indicate the recipient of their help that they have in mind: science, politicians, or, simply, society in general. Other categories have been mentioned by just a few respondents, e.g., that it is part of their routine or that the person recruiting them was nice. In Figure 2, the substantive amount of item nonresponse (almost 15%) and general unit nonresponse (around 10%) is also depicted. Results for other waves are not shown here, but do not differ much.

#### 4.3.2. Multivariate analysis: Survey motivation and panel attrition

Secondly, we will use the semi-automatically coded motivations for panel participation as a predictor variable in panel attrition analysis. As mentioned earlier, previous research (Brüggen et al., 2011) on participant motivation also often distinguishes between intrinsic and extrinsic motivation. An extrinsically motivated person would participate with the prospect of incentives or moral obligation. In contrast, an intrinsically motivated person would participate motivated by pleasure, curiosity, or interest, or they want to help and reveal their opinion. In the study by Brüggen et al. (2011), it was found that those with intrinsic motivations had the highest response rate, and those with extrinsic and self-focused motivation (incentives) had the lowest. According to this result, dropout should generally be higher among extrinsically motivated individuals, which is also consistent with the findings of Porst and Briel (1995). However, our goal is not to formally test these theories but rather to motivate our empirical demonstration.

**Figure 3:**
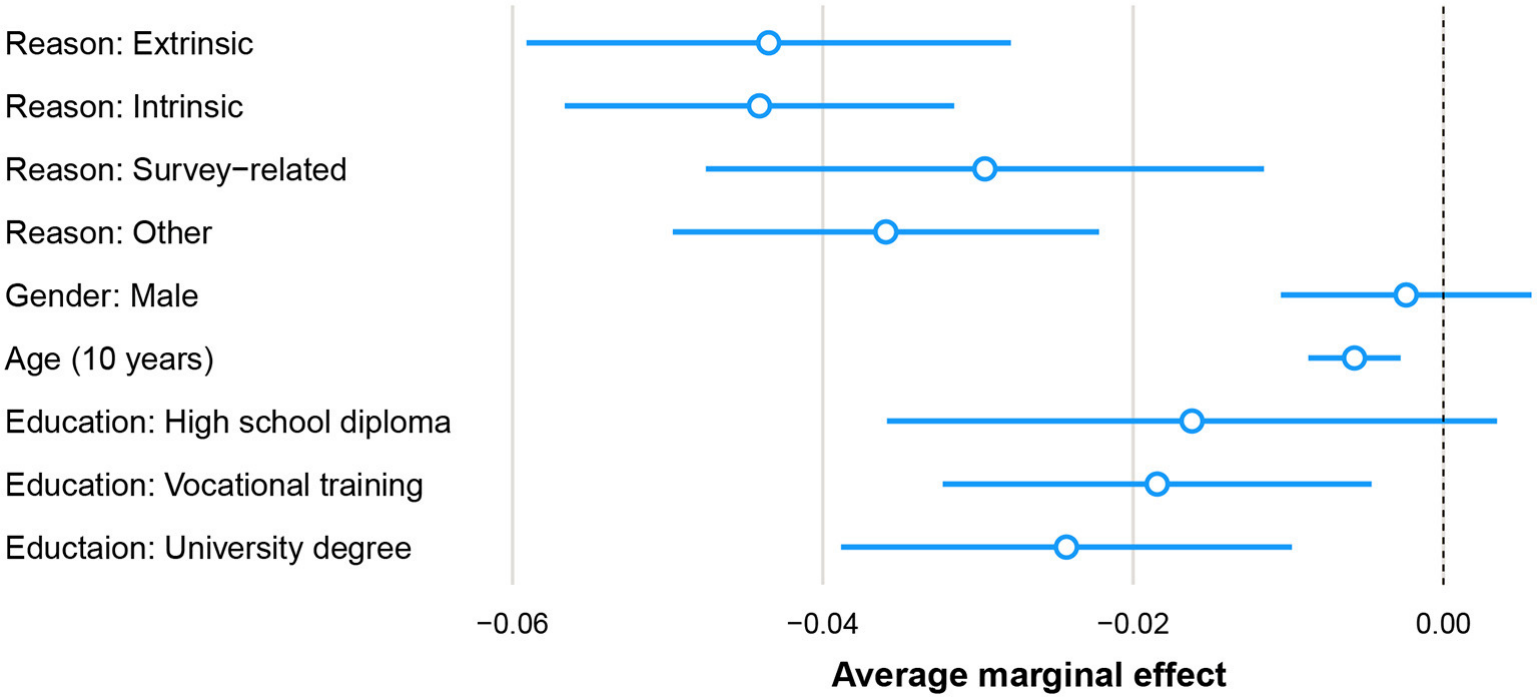
Logistic regression of panel dropout on independent variable most important participation reason, categorized in four broader categories “Extrinsic reasons,” “Intrinsic reasons,” and “Survey-related reasons,” and “Other reasons.” Reference category for reason: No reason given, reference category for gender: female, reference category for education: no formal education diploma. The AME for unit nonresponse is 0.4140 (SE 0.0183), not depicted since it is outside of the x-axis scale.

We take a look at the difference between the correlation of extrinsic and intrinsic motivation and panel dropout in the GESIS Panel between the years 2013 and 2018. We are, therefore, interested in the estimates of the model parameters for extrinsic and intrinsic in a logistic regression model with panel dropout as the dependent variable. This research question can be analyzed utilizing a discrete event-history model. A panelist of the GESIS Panel can drop out in two ways: either by requesting to be excluded from the panel management or by not participating in three subsequent waves of the panel. The data set used to estimate the model parameters consists of 6.031 panelists with 14.635 points of observation (2.4 years of being part of the panel on average). The data set is in person-period format, i.e., one row per person per period observed. The panel dropout indicator is set to 1 for the year in which the panelist dropped out of the panel (rows with dropout: 1,196, rows without dropout: 13,439). For periods thereafter, the panelist is not part of the sample anymore. We group the participation motivation categories that we presented earlier into broader categories, since the original categories include very small ones, potentially creating computational problems. We group them into the broader categories “Extrinsic reasons,” “Intrinsic reasons,” “Survey-related reasons,” and “Other reasons.” These broader categories were already indicated through the colors used in the barplot in Figure 2. Apart from the independent variable we are interested in, we also included other sociodemographic variables (gender, age, and education) and wave indicators as intercepts in our analysis. We present average marginal effects for easier interpretation than log odds ratios (Mood, 2010), the average marginal effect is the average change in probability when the independent variable increases by one unit. In Figure 3, we can see that persons who indicate either extrinsic or intrinsic reasons are much less likely to drop out in the year after than persons who do not answer the question. The difference between both, however, is small; we are therefore not able to replicate the findings by Porst and Briel (1995) and Brüggen et al. (2011).

## 5. Conclusion

With this article, we have made several contributions. First, we developed and presented a precise categorization scheme for reasons to participate in a panel survey, the GESIS Panel. We built on existing studies by Porst and Briel (1995) and Brüggen et al. (2011), but extended them significantly and tailored them to suit the requirements of panel studies better. While certain subcategories will probably differ in their magnitude for different surveys, our coding scheme can serve as a starting point for other survey researcher, just like Porst and Briel (1995) did serve as our—albeit much broader—starting point.

Second, we have also demonstrated that semi-automatic classification is a suitable tool to classify large sets of responses to open-ended questions in surveys. A potential application area of semi-automated classification are potentially other panel surveys with repeated questions or survey with response numbers >10, 000. Below this number, we estimate that the effort used to train and calibrate the semi-automated classification would—depending on the previous experience of researchers, of course—not be less than hand-coding the entire dataset. One also needs to keep in mind, that a certain number (at least 40 in our experience) of observations are needed for each category, a number that can be hard to achieve with small training sets or uneven distributions of categories. Some characteristics of the questionnaire design and the answers worked in our favor for semi-automatic classification: The answers were generally one-dimensional and short and concise by presenting the question with different fields for different reasons. Difficulties can, however, be encountered with answers that are too short and therefore ambiguous. This is not a problem that only occurs when using semi-automated classification, this is also a problem when using only hand-coding. As an example, we noticed several times that persons simply answered “Opinion” as a reason, it is however unclear to humans and machines alike whether the persons like to give their opinion or whether the person hopes that their opinion has an impact. Another limitation is also that a few categories (especially rare ones) were harder to predict than others, this might bias our analysis in a small way. To better handle spelling errors, we automatically corrected the answers with Hunspell before further pre-processing steps. We tested the use of sentence embeddings (Conneau et al., 2018) in addition to word counts, but in our case, we did not see any significant additional improvement in performance. Overall, however, the performance can be rated as very good.

Third, after semi-automatic classification, we used the newly generated measurements for descriptive analyses. Apart from a widely-shared sense among respondents that surveys are important, incentives and interest in the topics of the GESIS Panel are some of the most important motivators. Factors that have not been discussed prominently in literature are the wishes of many participants to help politicians and those in power understand what people think. It is also apparent that people enjoy sharing their opinion.

Fourth, we were also able to use the newly generated measurement with categories of participation in a simple analysis of (partial) correlations between survey motivation and panel attrition. While a full causal analysis would go beyond the scope of this article, we did notice that intrinsic or extrinsic motivations were clearly associated with less panel dropout than survey-related reasons (or item nonresponse and unit nonresponse). Again, this result can be seen as a good indicator of criterion validity. Other than Brüggen et al. (2011), however, we did not see any noticeable difference between intrinsic and extrinsic motivations regarding the association with panel dropout.

Several other research paths lead from here: regarding panel management, it may be worth tailoring cover letters to potential respondents to their motivations to increase participation rates further and lessen dropout. In addition, a thorough causal analysis would be important to examine the influence of motivation on the willingness to participate in surveys in more detail.

Another open question is how to enable further use of coded text responses or trained models. It would be helpful to have more comprehensive comparisons and explorations of general advantages and disadvantages of different semi-automated classification methods and algorithms. Another issue is the fact that answers to open-ended questions are not generally part of scientific use files since they can contain personal information, which would potentially allow the identification of respondents. A possible solution might be strategies that have been employed in other cases where data confidentiality has to be guaranteed: the creation of synthetic data sets (Drechsler, 2011) from the original data feature matrix. This could allow other researchers to train models and use these on their data, while at the same time minimizing disclosure risks.

## Data availability statement

The data analyzed in this study is subject to the following licenses/restrictions: We used data from the GESIS Panel (extended edition). The dataset can be accessed at the GESIS Leibniz Institute for the Social Sciences. https://search.gesis.org/research_data/ZA5664. Requests to access these datasets should be directed to https://search.gesis.org/research_data/ZA5664.

## Author contributions

A-CH and BW contributed to conception and design of the study. A-CH and KB hand-coded a subset of the text data together with student assistants. A-CH wrote the draft of the analysis script and wrote the draft of the manuscript. A-CH, PS, PC, and KB extended and performed the statistical analysis. BW contributed in-depth feedback. All authors contributed to manuscript revision and approved the submitted version.

## Conflict of interest

The authors declare that the research was conducted in the absence of any commercial or financial relationships that could be construed as a potential conflict of interest.

## Publisher's note

All claims expressed in this article are solely those of the authors and do not necessarily represent those of their affiliated organizations, or those of the publisher, the editors and the reviewers. Any product that may be evaluated in this article, or claim that may be made by its manufacturer, is not guaranteed or endorsed by the publisher.
