# Supplementary material for: The semi-automatic classification of an open-ended question on panel survey motivation and its application in attrition analysis
Source: Front Big Data. 2022 Aug 11;5:880554. doi: 10.3389/fdata.2022.880554 (PMC9403118; doi:10.3389/fdata.2022.880554)
Supplement: Supplementary file 1 [file Data_Sheet_1.pdf]

Supplementary Material: Semi-automatic classification of an open-ended question on survey motivation

1 EVALUATION OF THE TEST SET: CONFUSION MATRIX

	Interest	Curiosity	Learning	Tell opinion	Influence	Incentive	Fun	Routine	Help science	Help politicians	Help	Help, not further specified	Brevity	Professionalism	Recruiter	Recruitment	Importance in general	No reason/ Other
Interest	367	0	1	0	0	0	0	0	0	0	0	0	0	0	1	0	3	0
Curiosity	0	11	0	0	0	0	0	0	0	0	0	0	0	0	0	0	0	0
Education	0	0	5	0	0	0	0	0	0	0	0	0	0	0	0	0	2	0
Tell opinion	0	0	1	68	2	0	0	0	0	0	0	0	0	0	0	0	3	0
Influence	0	0	0	0	8	0	0	0	0	0	0	0	0	0	0	0	0	0
Incentive	0	0	0	0	0	534	0	0	0	0	0	0	0	0	0	1	2	0
Fun	0	0	0	0	0	0	111	0	0	0	0	0	0	0	0	0	1	0
Routine	0	0	0	0	0	0	0	19	0	0	0	0	0	0	0	0	0	0
Help science	0	0	0	0	0	0	0	0	19	0	1	1	0	0	0	0	2	0
Help politicians	0	0	0	0	0	0	0	0	0	4	0	1	0	0	0	0	0	0
Help society	1	0	0	0	1	0	0	0	0	0	25	0	0	0	0	0	2	0
Help, not further specified	0	0	0	0	1	0	0	0	2	0	1	7	0	0	0	0	1	0
Brevity	1	0	0	0	0	0	0	0	0	0	0	0	5	0	0	0	0	0
Professionalism	0	0	0	0	0	0	0	0	0	0	0	0	0	4	0	1	0	0
Recruiter	0	0	0	0	0	0	0	0	0	0	0	0	0	0	3	1	0	0
Recruiting	1	0	0	0	0	0	0	1	0	0	0	0	0	0	2	17	1	0
Importance in general	2	0	2	0	3	0	0	0	0	2	6	2	0	0	0	0	61	0
No reason/Other	1	0	1	1	1	4	1	2	0	0	0	1	1	3	0	1	21	107

2 PERFORMANCE INDICATORS

	Sensitivity	Specificity	Pos Pred Value	Neg Pred Value	Precision	Recall	F1	Prevalence	Detection Rate	Detection Prevalence	Balanced Accuracy
Interest	0.98	1.00	0.99	0.99	0.99	0.98	0.98	0.25	0.24	0.25	0.99
Curiosity	1.00	1.00	1.00	1.00	1.00	1.00	1.00	0.01	0.01	0.01	1.00
Education	0.50	1.00	0.71	1.00	0.71	0.50	0.59	0.01	0.00	0.00	0.75
Tell opinion	0.99	1.00	0.92	1.00	0.92	0.99	0.95	0.05	0.04	0.05	0.99
Influence	0.50	1.00	0.89	0.99	0.89	0.50	0.64	0.01	0.01	0.01	0.75
Incentive	0.99	1.00	0.99	0.99	0.99	0.99	0.99	0.36	0.35	0.35	0.99
Fun	0.99	1.00	0.99	1.00	0.99	0.99	0.99	0.07	0.07	0.07	1.00
Routine	0.86	1.00	1.00	1.00	1.00	0.86	0.93	0.01	0.01	0.01	0.93
Help science	0.90	1.00	0.83	1.00	0.83	0.90	0.86	0.01	0.01	0.02	0.95
Help politicians	0.67	1.00	0.80	1.00	0.80	0.67	0.73	0.00	0.00	0.00	0.83
Help society	0.76	1.00	0.86	0.99	0.86	0.76	0.81	0.02	0.02	0.02	0.88
Help in general	0.58	1.00	0.58	1.00	0.58	0.58	0.58	0.01	0.00	0.01	0.79
Brevity	0.83	1.00	0.83	1.00	0.83	0.83	0.83	0.00	0.00	0.00	0.92
Professionalism	0.57	1.00	0.80	1.00	0.80	0.57	0.67	0.00	0.00	0.00	0.79
Recruiter	0.50	1.00	0.75	1.00	0.75	0.50	0.60	0.00	0.00	0.00	0.75
Recruitment	0.81	1.00	0.77	1.00	0.77	0.81	0.79	0.01	0.01	0.01	0.90
Importance in general	0.60	0.99	0.77	0.97	0.77	0.60	0.67	0.07	0.04	0.05	0.79
No reason/Other	1.00	0.97	0.71	1.00	0.71	1.00	0.83	0.07	0.07	0.10	0.98

3 CODING SCHEME

German names for categories are given in parentheses. For the coding examples, a translation is given first, then the original German answer in brackets.

Interest (Interesse)

The respondent indicates that they are personally interested in the survey's topic, results, or other characteristics.

Examples taken from the survey:

- I find the survey interesting (Ich finde die Umfrage interessant)
- out of interest (aus Interesse)
- the questions interest me (mich interessieren die Fragen)
- the questions and various answers are also very interesting for me, because you don't normally think about such things (Die Fragen und verschiedenen Antworten sind für mich auch sehr interessant, weil man an sowas normal nicht denkt)
- I am interested in which topics are currently the focus of the survey (bin daran interessiert, welche Themen aktuell in den Fokus der Befragung gerückt werden)

Curiosity (Neugierde)

Respondents participate in the survey because they are curious about, for example, the current research, the results, or the topics of the survey.

Examples taken from the survey:

- Curiosity (Neugier)
- Curious to see what kind of surveys there are (Gespannt, was es so für Umfragen gibt)
- I'm curious every time to see what kind of questions there are this time (ich bin jedesmal neugierig, was es diesmal für Fragen gibt)

Learning (Weiterbildung)

Respondents take part in the survey because they want to educate themselves.

Examples taken from the survey:

- to think for myself on spec. topics (selbst Gedanken zu spez. Themen zu machen)
- own evaluation of my behavior (Eigene Bewertung meines Verhaltens)
- personal reflection (persönliche reflektion)
- input for thoughts (Gedankeninput)

Tell opinion (Meinung mitteilen)

Respondents take part in the survey because they want to share their opinion.

Examples taken from the survey:

- express my opinion (Meinungsäußerung)
- share my opinion (meine Meinung mitteilen)
- can express my opinion (kann meine Meinung äußern)
- contribute my opinion (Meine Meinung mit einbringen)

Influence (Einfluss nehmen)

Respondents have a clear desire to influence policy or research.

Examples taken from the survey:

- Hope to be able to influence politicians (Hoffe, Einfluss auf die Politiker nehmen zu können)
- Participate in political events (Teilhabe am politischen Geschehen)
- To improve the current situation (Zur Besserung der jetzigen Lage)
- Maybe to be able to change something (Vielleicht was verändern zu können)
- To help shape the future (Zukunft mitzugestalten)

Incentive (Incentive)

Participants take part in the survey because it is rewarded with money.

Examples taken from the survey:

- reward (Belohnung)
- well paid (gut bezahlt)
- premium of 5€ (Prämie von 5€)
- 5 euro (5 Euro)
- remuneration (Vergütung)

Fun (Spass)

Respondent participate in the survey because they enjoy it or because they feel some pleasure and comfort from participating.

Examples taken from the survey:

- Fun (Spaß)
- it's fun (es macht spass)
- I like doing surveys (Ich mache gerne Umfragen)
- fun answering the question (Spaß am Beantworten der Frage)
- because I like doing it (Weil ich es gerne mache)

Routine (Routine)

Respondent participate in the survey because it is a regular occurrence. They started it and want to remain consistent in their behavior.

- Regularity (Regelmäßigkeit)
- I was asked at the time and stuck with it (Ich wurde damals gefragt und bin dabei geblieben)
- Continuation of what was once started (Fortsetzung des einmal begonnenen)
- Participated once, then continued (einmal mitgemacht, dann weiter)

Dutifulness (Moralische Verpflichtung)

Respondents participate in the survey because they feel morally obligated to do so.

Examples taken from the survey:

- Obligation (Verpflichtung)
- I felt obligated (Ich fühlte mich verpflichtet)

- civic sense of duty (bürgerschaftliches Pflichtbewußtsein)
- civic obligation (Bürgerpflicht)

Help science (Wissenschaft helfen)

Respondents participate in the survey because they want to help science or emphasize the importance and significance of participation for science.

Examples taken from the survey:

- to help science (um der Wissenschaft zu helfen)
- research (Forschung)
- important to science (wichtig für die Wissenschaft)
- for social science research (für die sozialwissenschaftliche Forschung)

Help politicians (Politik helfen)

Respondents participate in the survey because they want to help politicians or to emphasize the importance and significance of participation in surveys for policy

Examples taken from the survey:

- would like to help political leaders (möchte Verantwortlichen aus der Politik helfen)
- because it is helpful to the federal government (weil es hilfreich für die Bundesregierung ist)

Help society (Gesellschaft helfen)

Respondents participate in the survey because they want to help the society or underline the importance of survey participation for society (or economy).

Examples taken from the survey:

- important for the society (wichtig für die Gesellschaft)
- for the society (für die Gesellschaft)
- my contribution to the society (mein beitrag zur Gesellschaft)
- surveys are important for the society (Umfragen sind für Gesellschaft wichtig)
- it is important for the general public (Es ist wichtig für das Allgemeinheit)

Help, not further specified (Helfen, unspezifisch)

Respondents participate in the survey because they want to help but do not further specify to whom this help is directed.

Examples taken from the survey:

- would like to help (möchte helfen)
- to do others a favor (um anderen einen Gefallen zu tun)
- out of helpfulness (Aus Hilfsbereitschaft)
- to help (um zu helfen)

Survey-related reasons

Brevity (Kürze)

Respondents participate in particular because of the brevity of the questionnaire.

Examples taken from the survey:

- Have that little bit of time to spare (Habe das bisschen Zeit übrig)
- goes quickly (geht zügig)
- NOT LONG (NICH LANG)
- surveys don't take that long (die Umfragen dauern nicht so lange)
- short completion time (kurze Ausfüllzeit)

Anonymity (Anonymität)

Respondents participate in the survey because of the promised anonymity.

Examples taken from the survey:

- anonymity (Annonymitaet)
- anonymous (anonym)
- anonymized data collection (anonymisierte Datenerhebung)
- everything remains anonymous (alles bleibt anonym)

Professionalism (Seriosität)

Respondents participate since they trust the GESIS Institute and think it is a respectable institution.

Examples taken from the survey:

- respectable interviewer (Seriöser Fragesteller)
- because I believe GESIS is respectable, I don't take part in any other survey (weil ich glaube GESIS ist serisös,nehme sonst an keiner Umfrage teile)
- respectable appearance of GESIS (Seriöses Auftreten von GESIS)
- respectable institute (seriöses Institut)

Recruitment (Rekrutierung)

Respondents participate in the survey because they were sampled and asked to participate.

Examples taken from the survey:

- I was asked (man hat mich gefragt)
- was asked to do this (wurde darum gebeten)
- asking if I wanted to participate (Anfage, ob ich teilnehmen möchte)

Recruiter (Rekrutierer)

Respondents participate in the interview because of the characteristics of the recruiter or the recruitment interview. In addition to the recruitment, there is a reference to the recruiter or the recruitment interview in this class.

Examples taken from the survey:

- I was persuaded (Bin überredet worden.)
- I was asked personally in a pleasant way (bin persönlich auf angenehme Art gefragt worden)
- I was contacted in a pleasant way (Bin angenehm kontaktiert worden)
- The personal interview was very interesting (Das persönliche vorgrspräch war sehr interessant)

Further survey-related reasons

Respondents participate because of the characteristics of the panel. Interest and curiosity regarding the questions (e.g., interesting questions) are not put into this class but in the interest class.

Examples taken from the survey:

- good questions (gute Fragen)
- social surveys (Gesellschaftsumfragen)
- flexible online completion (flexibel online ausfüllbar)
- meaningful survey, not for advertising purposes (Sinnvolle Umfrage, nicht für Werbezwecke)
- diversified (abwechslungsreich)

Other categories

Importance in general (Bedeutung im Allgemeinen)

Examples taken from the survey:

- important (wichtig)
- environment (Umwelt)
- Participation is important for policy (Teilnahme ist wichtig für die Politik)
- I find it important and good (ich finde es wichtig und gut)

No reason/Other (Kein Grund/Other)

Respondents explicitly say that they have no reason to participate. Answers that cannot be interpreted or put into one of the other categories are classified in this class.

Examples taken from the survey:

- no important reason (kein wichtiger Grund)
- no reasons not at all (Keine Gründe nicht überhaupt nicht)
- I have no reasons (Ich habe keine Gründe)
- my parents force me (Meine Eltern zwingen mich)
- questions (Fragen)

4 LOGISTIC REGRESSION COEFFICIENTS

Table S1. Logistic Regression Results

	Dependent variable:
	Dropout
Year 1	-1.604***
	(0.182)
Year 2	-1.425***
	(0.182)
Year 3	-1.624***
	(0.185)
Year 4	-1.762***
	(0.189)
Reason: Extrinsic	-0.759***
	(0.154)
Reason: Intrinsic	-0.774***
	(0.100)
Reason: Other	-0.586***
	(0.112)
Reason: Survey-related	-0.458***
	(0.154)
Reason: Unit Nonresponse	2.387***
	(0.102)
Gender: Male	-0.039
	(0.067)
Education: High school diploma	-0.240
	(0.151)
Education: Completed vocational training	-0.276***
	(0.100)
Education: University degree	-0.375***
	(0.109)
Age in 10 years	-0.094***
	(0.025)
Log Likelihood	-3,380.863
Akaike Inf. Crit.	6,789.726

Table S2. Logistic regression of panel dropout on independent variable most important participation reason, categorized in four broader categories Extrinsic reasons, Intrinsic reasons, Survey-related reasons. Other reasons. Reference category for reason: No reason given, reference category for gender: female, reference category for education: no formal education diploma/still in education. *Note:* ***p<0.01
